# From Waste to Resource: Nutritional and Functional Potential of Borlotto Bean Pods (*Phaseolus vulgaris* L.)

**DOI:** 10.3390/antiox14060625

**Published:** 2025-05-23

**Authors:** Antonella Smeriglio, Martina Imbesi, Mariarosaria Ingegneri, Rossana Rando, Manuela Mandrone, Ilaria Chiocchio, Ferruccio Poli, Domenico Trombetta

**Affiliations:** 1 Department of Chemical, Biological, Pharmaceutical and Environmental Sciences (CHIBIOFARAM), University of Messina, Viale Ferdinando Stagno d’Alcontres 31, 98166 Messina, Italy; martina.imbesi@studenti.unime.it (M.I.); mariarosaria.ingegneri@unime.it (M.I.); domenico.trombetta@unime.it (D.T.); 2 Department of Biomedical, Dental, Morphological and Functional Image Sciences (BIOMORF), University of Messina, Via Giovanni Palatucci, 98168 Messina, Italy; rossana.rando@unime.it; 3 Department of Pharmacy and Biotechnology (FaBit), Alma Mater Studiorum, University of Bologna, Via Irnerio 42, 40126 Bologna, Italy; manuela.mandrone2@unibo.it (M.M.); ilaria.chiocchio2@unibo.it (I.C.); ferruccio.poli@unibo.it (F.P.)

**Keywords:** *Phaseolus vulgaris* L. *var.* borlotto, bean pod, primary metabolites, ^1^H-NMR profiling, secondary metabolites, LC-DAD-ESI-MS/MS, polyphenols, antioxidant activity, anti-inflammatory activity, circular economy

## Abstract

Borlotto bean pods, a by-product of *Phaseolus vulgaris* processing, represent a promising yet underexplored source of bioactive compounds. This study aimed to characterize the nutritional composition, phytochemical profile, and biological properties of a food-grade extract obtained from borlotto bean pods (BPE). Nutritional parameters were assessed using standard AOAC methods, while primary and secondary metabolites were identified and semi-quantified via ^1^H-NMR and LC-DAD-ESI-MS/MS. Antioxidant activity was evaluated through six complementary assays: DPPH, TEAC, FRAP, ORAC, ferrous ion-chelating activity, and β-carotene bleaching inhibition. Anti-inflammatory potential was assessed in vitro by evaluating the inhibition of bovine serum albumin (BSA) denaturation and protease activity. BPE showed significant antioxidant capacity across all assays, indicating both hydrogen atom transfer and electron transfer mechanisms, along with metal chelation and lipid peroxidation inhibition. Additionally, BPE inhibited protein denaturation and protease activity in a concentration-dependent manner. These results highlight the potential of borlotto bean pods as a sustainable source of nutritionally and functionally relevant compounds. Future studies should focus on the bioavailability of active constituents, formulation into delivery systems, and in vivo validation to support potential nutraceutical applications.

## 1. Introduction

Legumes are an essential component of most diets worldwide [1]. Beans, chickpeas, lupins, peas, broad beans and lentils account for 80% of global legume production [2]. However, 25% of these vegetables are discarded during harvesting and processing due to the failure to meet production quality standards. These waste products, which include the seed tegument, broken seeds, pods, washing and blanching water, and pomace, nevertheless contain a high concentration of nutritional and health-promoting molecules [1,3]. For instance, broken beans (which constitute about 0.025% of the total annual production) are often discarded, even though the seed tegument is rich in polyphenols with strong antioxidant properties [4]. These waste products could be used as they are or could also be processed into techno-functional foods such as flour, snacks, gluten-free products, or protein foods for vegetarians and vegans or for those people with intolerance, given their low allergenic power [1,5].

It has long been known that regular consumption of beans, which are very rich in protein, fiber, and polyphenols, helps regulate certain molecular markers related to non-communicable diseases such as cancer, type 2 diabetes, hypertension, and obesity. The consumption of these foods, therefore, or the use of their by-products to produce techno-functional ingredients or plant-complexes for use in nutraceuticals, can certainly be useful strategies to provide not only nutritional but also health benefits [5,6,7].

Beans are the most widely consumed legumes globally, with world production of 27.6 million tons per year, of which approximately 71% is for human consumption [5,8]. These legumes are rich in essential amino acids, particularly leucine, phenylalanine and lysine, phenolic compounds, and fiber in the outer layers of the seed, with a low content of saturated fat [9]. Consumption of beans is associated with a significant reduction in LDL-cholesterol (~20%) as well as a decrease in body weight by approximately 4% compared to a diet in which beans are not present [7]. The cotyledons of the seed contain high concentrations of phenolic acids (ferulic, protocatechic, aldaric, p-coumaric) and flavonoids (kaempferol, quercetin, myricetin, catechin, epicatechin), including anthocyanins (delphinidin, petunidin, malvidin, 3-O-glucosides) that are particularly abundant in borlotto bean and black bean. However, these compounds are often more abundant in the seed tegument and pod, parts that are often discarded during processing and that could instead play an important role as a plant source for the recovery of these active ingredients with well-documented bioactivities [10,11,12].

The use of bean flour has already been successfully explored in bars, biscuits, pasta, and tortillas, but a circular-economy approach to recovering the same nutritional and health-promoting molecules found in the edible part of the bean from the waste products obtained during its processing has never been considered [5]. This could help not only to reduce waste, with a not inconsiderable economic benefit for the producing companies that often must dispose of these by-products as special waste, but also to significantly reduce the environmental impact, making the bean and legume supply chain in general more eco-sustainable.

Despite the known nutritional potential of common bean seeds, the valorization of borlotto bean pods—a major industrial by-product—remains unexplored. This study addresses this gap by providing a comprehensive characterization of this underutilized matrix. Several studies have investigated the phenolic composition and antioxidant activity of legume-derived by-products, such as soybean hulls, black bean seed coats, and pea pods, highlighting their potential as sources of bioactive compounds [13,14]. However, despite the extensive cultivation and consumption of *P. vulgaris*, borlotto bean pods remain largely unexplored in this context. To date, no comprehensive characterization of their polyphenolic profile or antioxidant and anti-inflammatory properties has been reported. Therefore, valorizing borlotto bean pods could represent a promising opportunity for the recovery of high-value phytochemicals from agro-industrial waste. In view of this, the goal of this study was first to investigate the nutritional properties of the borlotto bean pod, the main waste in terms of biomass obtained from its processing. In parallel, a food-grade polyphenol-rich extract was obtained and analyzed, both phytochemically, by characterizing its primary and secondary metabolites, and biologically, by assessing its antioxidant and anti-inflammatory properties, to explore its potential for nutraceutical or functional applications under more controlled and concentrated conditions.

## 2. Materials and Methods

### 2.1. Chemicals

The following chemical reagents were purchased from Merck KGaA (Darmstadt, Germany): ultrapure water, hydrogen peroxide (H_2_O_2_, 30% *v*/*v*), nitric acid (HNO_3_, 65% *v*/*v*), sodium standard for ICP (1 g/L Na in 2% nitric acid), 2(N-morpholino) ethanesulfonic acid (MES), tris(hydroxymethyl)aminomethane (TRIS), Megazyme Total Dietary Fibre kit (α-amylase, protease and amyloglucosidase), ethanol (C_2_H_6_O), acetone (C_3_H_6_O), hydrochloric acid (HCl), sodium hydroxide (NaOH), phenol (C_6_H_6_O), sulphuric acid (H_2_SO_4_), catalyst in tablets for Kjeldahl mineralization, boric acid (H_3_BO_3_), chloroform (CHCl_3_), methanol (CH_3_OH), sodium chloride (NaCl), potassium hydroxide (KOH), *n*-heptane (C_7_H_16_), 37 component FAME mix in dichloromethane, Folin–Ciocalteu reagent, sodium carbonate (Na_2_CO_3_), gallic acid, rutin, sodium nitrite (NaNO_2_), aluminium chloride (AlCl_3_) catechin (C_15_H_16_O_6_), vanillin (C_8_H_8_O_3_), absolute ethanol, iron(II) sulphate (FeSO_4_), cyanidin chloride, 2,2-diphenyl-1-picrylhydrazyl radical (DPPH), 2-2′-azino-bis-(3-ethyl-benzothiazolin-6-sulphonic acid) (ABTS), ammonium persulphate, potassium peroxydisulphate, 6-hydroxy-2,5,7,8-tetramethylchromane-2-carboxylic acid (trolox), 2-2-azobis (2-methylpropionamide) dihydrochloride (AAPH), 2,4,6-tris(2-pyridyl)-s-triazine (TPTZ), EDTA, iron(II) chloride (FeCl_2_), ferrozine, β-carotene, butylated hydroxytoluene (BHT), Tween-40, linoleic acid, bovine serum albumin (BSA) without fatty acids, diclofenac sodium, Tris-HCl, casein, and trypsin type IX-S from porcine pancreas. Deuterium oxide (D_2_O, 99.90% D) and deuterated methanol (CD_3_OD, 99.80% D) were purchased from Eurisotop (Cambridge Isotope Laboratories, Inc, France). The sodium salt of standard 3-(trimethylsilyl)-propionic acid-2,2,3,3-d4 (TMSP), anhydrous dibasic sodium phosphate, anhydrous monobasic sodium phosphate, and all other chemicals and solvents used for ^1^H-NMR analysis were purchased from Sigma-Aldrich Co. (St. Louis, MO, USA).

Reference standards (purity ≥ 98%) used for polyphenols characterization were purchased from Extrasynthese (Genay, France) and Merck KGaA (Darmstadt, Germany).

### 2.2. Sample Preparation

Borlotto beans (*Phaseulus vulgaris* L. var. borlotto) were purchased from a local farmer in Messina (Sicily, Italy).

The pods were manually removed from the seeds, cut into small pieces, and cryo-powdered in liquid nitrogen using a blade mill (IKA^®^ A11, IKA^®^-Werke GmbH & Co., Ltd. KG, Staufen, Germany) to preserve the nutritional characteristics and to retain the phytochemical composition of the original matrix. The powdered sample thus obtained was used for the characterization of the nutritional profile and to obtain the food-grade extract.

### 2.3. Nutritional Composition

To assess the nutritional composition of the bean pods, three independent batches were each analyzed in triplicate (*n* = 3). Energy, moisture, total fiber, sugars, ash, proteins, and fats were determined [15].

For fatty acid profiling, 4 g of the bean pod sample was extracted twice with 40 mL of a chloroform/methanol mixture (2:1, *v*/*v*) and centrifuged at 7000 rpm for 15 min at room temperature (RT) following the addition of 10 mL of 0.73% NaCl solution. The resulting supernatants were evaporated to dryness using a rotary evaporator (R-210, BUCHI, Cornaredo, Italy). Trans-methylation was performed using *n*-heptane and a methylating mixture composed of methanol and potassium hydroxide (2N) to obtain fatty acid methyl esters (FAMEs), which were subsequently analyzed by gas chromatography coupled with a flame ionization detector (GC-FID, Dani Master GC, Dani Instrument, Milan, Italy).

Separation was achieved using a ZB-Wax column (30 m × 0.25 mm × 0.25 µm; Phenomenex, Torrance, CA, USA) with helium as the carrier gas (30 cm/s). Samples were injected at 240 °C in split mode (50:1). The oven temperature was scheduled to start at 50 °C for 2 min, followed by a ramp of 3 °C/min up to 240 °C, which was then held for 15 min. The detector temperature was maintained at 240 °C. Data acquisition and management were performed using Clarity Chromatography v 4.0.2 software (DataApex, Prague, Czech Republic). Detected compounds were identified based on retention time matching with a Supelco 37-component FAME standard mixture. Quantitative analysis was conducted by normalizing peak areas.

Sodium concentration was determined using inductively coupled plasma mass spectrometry (ICP-MS) following mineralization of the bean pod samples. Specifically, 0.5 g of accurately weighed sample was digested with 8 mL of 65% HNO_3_ and 2 mL of 30% H_2_O_2_ in Teflon vessels. Mineralization was carried out by increasing the temperature to 180 °C in 15 min at a power of 1000 W, followed by holding this temperature for an additional 15 min at the same power using an Ethos One microwave digestion system (Milestone, Milan, Italy).

Blanks were prepared using the same procedure, but without sample addition. After digestion, the samples and blank solutions were diluted to a final volume of 25 mL with MilliQ water. The solutions were then filtered through a 0.45 µm filter and analyzed using an ICP-MS iCAP-Q (Thermo Fisher Scientific, Waltham, MA, USA) equipped with a Cetac-ASX520 autosampler.

The ICP-MS operating parameters were as follows: 1500 W generator power, 2 °C atomization chamber temperature, argon gas flow rates of 15/0.9/1.1 L/min (plasma/auxiliary/carrier), sample introduction flow rate of 1.0 mL/min, helium gas flow rate of 4.0 mL/min (collision cell), vacuum pressure below 7.5 × 10^−7^ Pa, and interface pressure of 5.3 × 10^−2^ Pa.

Quantitative determination of sodium was performed by constructing a calibration curve using standard solutions at five different concentration levels, prepared by diluting a 1000 mg/L sodium standard stock solution with ultra-pure water.

### 2.4. Preparation of Food-Grade Extract

Fifteen grams of frozen bean pod powder were extracted with 150 mL of 96% ethanol/H_2_O (70:30, *v*/*v*). The mixture was sonicated in an ice-cold bath using an ultrasonic probe sonicator operated at 200 W power and 30% amplitude for 10 min, followed by maceration under continuous stirring in darkness at RT for 2 h. After decantation, the supernatant was separated and filtered (Whatman No. 1, GE Healthcare). To ensure thorough recovery, the extraction procedure was repeated two additional times. The combined supernatants were evaporated to dryness using a rotary evaporator (Hei-VAP Core HL-G1, Heidolph, Schwabach, Germany), resulting in an average extraction yield of 1.58%. The obtained bean pod extract (BPE) was subsequently placed overnight in a vacuum desiccator containing anhydrous sodium sulphate. Prior to phytochemical characterization and subsequent analyses, the dried extract was freshly re-dissolved in a 96% ethanol/H_2_O (70:30, *v*/*v*) solution.

### 2.5. ^1^H-NMR Profiling

Ten milligrams of the extract were dissolved in 1 mL of a 1:1 mixture of phosphate buffer (90 mM, pH 6.0) prepared in D_2_O (containing 0.1% TMSP) and CD_3_OD. Subsequently, 700 μL of the solution were transferred into NMR tubes. ^1^H NMR and heteronuclear two-dimensional correlation spectra (HMBC and HSQC) were acquired at 25 °C using a Bruker Avance Ascend 600 MHz spectrometer equipped with autosamplers and a Prodigy cryoprobe.

For ^1^H NMR profiling, the spectrometer operated at a proton frequency of 600.13 MHz, with CD_3_OD used as the internal lock. The ^1^H NMR spectra were acquired over 46 scans with a relaxation delay (RD) of 2 s and a spectral width of 9595.8 Hz (corresponding to δ 16.0), resulting in a total acquisition time of approximately 4 min. A pre-saturation sequence (PRESAT) was applied to suppress the residual water signal at δ 4.83.

All spectra were manually phased, baseline-corrected, and referenced to the internal standard, trimethylsilyl propionic acid sodium salt (TMSP, δ 0.0), which also served as the reference compound for semiquantitative analysis. The spectral region between δ 0.0 and 10.0 was segmented into integrated buckets of equal width (δ 0.04) and normalized to the total spectral area using MestReNova 12 software (Mestrelab Research, Santiago di Compostela, Spain).

### 2.6. Phytochemical Screening

#### 2.6.1. Total Phenolic Content

Total phenolic content in the extract was quantified using the Folin–Ciocalteu method as described by Ingegneri et al. [16]. Briefly, 10 µL of BPE (10–40 mg/mL) was mixed 1:10 (*v*/*v*) with Folin–Ciocalteu reagent. After adding 90 μL Milli-Q water, samples were vortexed and incubated in the dark for 3 min. Subsequently, 100 μL of 10% Na_2_CO_3_ solution was added, and the mixture was incubated at RT for 1 h, with vortexing every 10 min. Absorbance was measured at 785 nm using a Multiskan™ GO UV-vis plate reader (Thermo Scientific, Waltham, MA, USA). Gallic acid (0.075–0.6 mg/mL) served as the reference standard, and a 96% ethanol/H_2_O (70:30, *v*/*v*) solution was used as a blank. Data were expressed as mg of gallic acid equivalents (GAE)/100 g of dry extract (DE).

#### 2.6.2. Total Flavonoids

Flavonoids were determined using rutin as a reference standard (0.125–1 mg/mL in CH_3_OH) according to Ingegneri et al. [16]. Briefly, 50 µL of sample solution (10–40 mg/mL) or 96% ethanol/H_2_O (70:30, *v*/*v*) as blank were mixed with 450 µL of Milli-Q water, followed by the addition of 30 µL of 5% NaNO_2_. After incubation at RT for 5 min, 60 µL of a 10% AlCl_3_ solution was added, and the mixture was left to stand for an additional 6 min. Finally, 200 µL of 1 M NaOH and 210 µL of MilliQ water were added. Samples were vortexed thoroughly, and absorbance was measured at 510 nm using a UV-1601 spectrophotometer (Shimadzu, Kyoto, Japan). Flavonoid content was expressed as mg rutin equivalents (RE)/100 g DE.

#### 2.6.3. Vanillin Index

Flavan-3-ols were quantified using the vanillin index assay as described by Ingegneri et al. [17]. Briefly, 2.0 mL of BPE (20 mg/mL), previously diluted in 0.5 M H_2_SO_4_, was loaded onto a pre-conditioned Sep-Pak C18 cartridge (Waters, Milan, Italy), washed with 2.0 mL of 5.0 mM H_2_SO_4_, and subsequently eluted with 5 mL of CH_3_OH. The eluate (1 mL) was then combined with 6.0 mL of 4% vanillin methanol solution and incubated at 20 °C for 10 min, followed by the addition of 3 mL of HCl and incubation in the dark for 15 min. Absorbance was measured at 500 nm using the same UV-vis spectrophotometer and blank mentioned in Section 2.6.2. Using catechin as the reference standard (125–500 µg/mL), results were reported as mg CE per 100 g of dry extract (DE).

#### 2.6.4. Proanthocyanidins

Proanthocyanidins were determined according to Ingegneri et al. [17]. Briefly, 2 mL of BPE (40 mg/mL) diluted in 0.05 M H_2_SO_4_ was loaded onto a pre-conditioned Sep-Pak C18 cartridge (Waters, Milan, Italy). The sample was eluted with 3 mL CH_3_OH and collected in a 100 mL round-bottom flask containing 9.5 mL absolute ethanol. Subsequently, 12.5 mL of a FeSO_4_·7H_2_O solution in hydrochloric acid (300 mg/L) was added, and the mixture was refluxed for 50 min. After cooling, absorbance was measured at 550 nm using the same spectrophotometer and blank described in Section 2.6.2. Proanthocyanidins were quantified by subtracting the absorbance of a similarly prepared sample kept cold (basal anthocyanin content). Results, calculated as five times the concentration of cyanidin formed, using the molar extinction coefficient of cyanidin chloride (ε = 34,700), were expressed as mg cyanidin chloride equivalents (CyE)/100 g DE.

### 2.7. Polyphenol Profile by LC-DAD-ESI-MS/MS Analysis

The polyphenol profile of BPE was characterized using LC-DAD-ESI-MS/MS according to Danna et al. [18]. Chromatographic separation was performed at 25 °C using a Luna Omega PS C18 column (150 mm × 2.1 mm, 5 µm; Phenomenex, Torrance, CA, USA), with 0.1% formic acid (solvent A) and methanol (solvent B) as mobile phases. The gradient profile was programmed as follows: 0% B (0–3 min), 3% B (3–9 min), 12% B (9–24 min), 20% B (24–33 min), 30% B (33–43 min), 50% B (43–66 min), 60% B (66–81 min), 0% B (81–86 min), with a final 4 min equilibration. Five microliters of BPE were injected, and UV-vis spectra were acquired between 190 and 600 nm. Chromatograms were recorded at 260, 292, 330, 370, and 520 nm to cover all polyphenol classes. Mass spectrometry was conducted using an Agilent 6320 ion trap instrument (Agilent Technologies, Santa Clara, CA, USA) operating in both negative and positive electrospray ionization (ESI) modes. Instrumental parameters were set as follows: capillary voltage, 3.5 kV; nebulizer gas (N_2_) pressure, 40 psi; drying gas temperature, 350 °C; drying gas flow rate, 9 L/min; and skimmer voltage, 40 V. Mass spectra were acquired in full-scan mode over an *m*/*z* range of 90–1000, with a fragmentation energy of 1.2 V applied for MS/MS analyses. Data acquisition and processing were performed using Agilent ChemStation software (version B.01.03) and Agilent Trap Control (version 6.2). Identification of analytes was based on retention time, UV–Vis, and MS/MS spectral comparison with HPLC-grade reference standards, when available, as well as literature reports, and open-access databases such as SpectraBase^®^, PhytoHub, ReSpect, MassBank, and PubChem. The abundance of polyphenols in total ion current chromatograms was expressed as ion peak intensity.

### 2.8. Antioxidant and Anti-Inflammatory Assays

The biological activity of BPE was evaluated using various in vitro colorimetric assays based on different mechanisms and reaction environments. Results were expressed as the concentration required to inhibit 50% of the oxidative or inflammatory activity (IC_50_, µg/mL), with corresponding 95% confidence limits (C.L.) calculated using the Litchfield and Wilcoxon method with PHARM/PCS software (version 4; Consulting, Wynnewood, PA, USA). Reported concentration ranges refer to final concentrations of samples and standards within the reaction mixtures.

#### 2.8.1. DPPH Assay

Briefly, 3.75 µL of BPE (250–2000 µg/mL) was mixed with 150 µL DPPH methanol solution (10^−4^ M) [16]. After stirring (10 s), sample was incubated in darkness at RT for 20 min. Absorbance was recorded at 517 nm against a blank (96% ethanol/H_2_O, 70:30, *v*/*v*) using the UV-vis reader described in Section 2.6.1. Trolox (2.5–20.0 µg/mL) was employed as the reference standard.

#### 2.8.2. TEAC Assay

A mixture of ABTS^+^ (1.7 mM) and K_2_S_2_O_8_ (4.3 mM) at a 1:5 *v*/*v* ratio was incubated at RT in darkness for 12 h and diluted to obtain an absorbance of 0.7 ± 0.02 at 734 nm [16]. Ten microliters of BPE (125–1000 µg/mL) or trolox standard (0.625–10 µg/mL) were added to 200 µL of the ABTS solution and incubated for 6 min at RT. The absorbance was recorded at 734 nm with the same UV-vis reader described in Section 2.6.1.

#### 2.8.3. FRAP Assay

Briefly, 10 µL of BPE (250–2000 µg/mL) or Trolox standard (1.25–10 µg/mL) was mixed with 200 µL of FRAP reagent, pre-warmed to 37 °C, consisting of 10 mM TPTZ in 40 mM HCl, 20 mM FeCl_3_·6H_2_O, and 300 mM acetate buffer (pH 3.6) [16]. Following a 4 min dark incubation at RT, the absorbance was recorded at 593 nm using the instrument described in Section 2.6.1.

#### 2.8.4. ORAC Assay

Twenty microliters of BPE (0.78–6.25 µg/mL) or Trolox standard (0.25–2 µg/mL) were added to 120 µL fluorescein (117 nM) [16]. After 15 min of incubation at 37 °C, 60 µL of 40 mM AAPH was added, and fluorescence was monitored every 30 s for 90 min (excitation: 485 nm; emission: 520 nm) using a FLUOstar Omega microplate reader (BMG LABTECH, Ortenberg, Germany).

#### 2.8.5. Iron-Chelating Activity

Iron-chelating activity (ICA) was evaluated using the ferrozine assay according to Ingegneri et al. [17]. Briefly, 25 µL FeCl_2_·4H_2_O (2 mM) was mixed with 50 µL BPE (20–160 µg/mL) or EDTA standard (1.75–14 µg/mL). Following a 5 min incubation at RT, 50 µL ferrozine (5 mM) and deionized water (final volume 1.5 mL) were added. After vortexing and a 10 min incubation, absorbance was measured at 562 nm using the instrument described in Section 2.6.1.

#### 2.8.6. β-Carotene Bleaching (BCB) Assay

The BCB assay followed Ingegneri et al. [17] with modifications. Briefly, 200 µL BPE (12.5–100 µg/mL), BHT standard (1 mg/mL), or blank (96% ethanol/H_2_O, 70:30, *v*/*v*) was mixed with 5 mL β-carotene emulsion (250 µL β-carotene solution, 1 mg/mL ethyl acetate; 4 µL linoleic acid; 40 µL Tween-40). A β-carotene-free emulsion was used as negative control. Samples were incubated at 50 °C for 2 h, and absorbance at 470 nm was recorded every 20 min using the instrument described in Section 2.6.1.

#### 2.8.7. Heat-Induced Bovine Serum Albumin (BSA) Denaturation Assay

BPE (125–1000 µg/mL) inhibitory activity on BSA denaturation was assessed as per Ingegneri et al. [17]. BPE was mixed with 0.4% BSA solution and PBS (pH 5.3) at a 4:5:1 ratio. Diclofenac sodium (3.0–24.0 µg/mL) was the reference standard. Absorbance at 595 nm was measured at baseline and after 30 min of incubation at 70 °C in a shaking water bath, using the UV–Vis reader described in Section 2.6.1.

#### 2.8.8. Protease-Inhibitory Activity (PIA)

PIA was determined as described by Ingegneri et al. [17]. Briefly, 200 µL of BPE (50–400 µg/mL) or diclofenac sodium (5–80 µg/mL) was mixed with 12 µL of trypsin (10 µg/mL), 188 µL of Tris-HCl buffer (20 mM, pH 7.5), and 200 µL of casein solution (0.8%). The mixture was incubated at 37 °C for 20 min. The enzymatic reaction was terminated by adding 400 µL of 2 M perchloric acid, followed by centrifugation at 3500× *g* for 10 min. The absorbance of the resulting supernatant was measured at 280 nm using the spectrophotometer described in Section 2.6.2.

### 2.9. Statistical Analysis

Each assay was performed in triplicate (*n* = 3) with five analytical replicates. Statistical significance (*p* < 0.05) was assessed using one-way ANOVA followed by the Student-Newman-Keuls post hoc test (SigmaPlot 12.0; Systat Software Inc., San Jose, CA, USA).

## 3. Results

### 3.1. Nutritional Characterization

The bean pod was simply powdered using a blade mill with liquid nitrogen to preserve its nutritional, phytochemical, and organoleptic properties. It was then subjected to a nutritional characterization by evaluating the calorific value as well as moisture, fat, protein, carbohydrate (sugars and total fiber), ash, and sodium content. The results, which were expressed as g/100 g of fresh weight (FW) and both as Kcal and KJ for calorific value, are shown in Table 1.

These results indicate that the matrix under investigation represents a valuable source of nutritionally relevant compounds. Not only does it have a low caloric value, but it also contains a good percentage of carbohydrates (~20%), half of which are fibers. It has a very low lipid content and an important sodium content which, while it may be useful in deficiency states as it can modulate the body’s acid-base balance, water balance, osmotic pressure, and nerve function, which certainly deserves attention in those subjects who must maintain a dietary regime poor in this microelement.

The lipid fraction was further investigated by characterizing the FAME by GC-FID. As can be seen from Table 2, despite its low lipid content, the bean pod is a valuable source of monounsaturated fatty acids (51.69 ± 2.02), followed by saturated (35.13 ± 1.13) and polyunsaturated (13.18 ± 1.10) FAME. Oleic acid, a monounsaturated fatty acid, was identified as the most abundant component (47.56 ± 2.07), followed by the saturated fatty acid palmitic acid (23.47 ± 1.30), and the polyunsaturated fatty acid linoleic acid (10.45 ± 1.02).

### 3.2. ^1^H NMR Profiling

**Figure 1:**
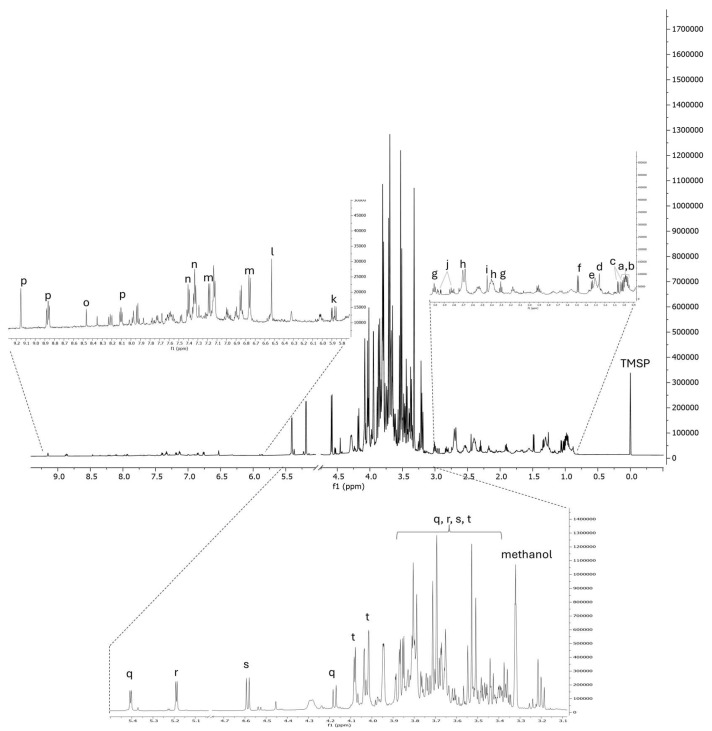
^1^H-NMR profile of BPE. a = isoleucine; b = leucine; c = valine; d = fatty acids; e = threonine; f = alanine; g = GABA; h = citric acid; i = succinic acid; j = asparagine; k = flavonoids; l = fumaric acid; m = tyrosine; n = phenylalanine; o = formic acid; p = trigonelline; q = sucrose; r = α-glucose; s = β-glucose; t = fructose. TMSP = 3-(trimethylsilyl)-propionic acid-2,2,3,3-d4 (internal standard).

Following nutritional characterization, the bean pod was subjected to hydroalcoholic extraction by ultrasound-assisted maceration to obtain a bean pod food-grade extract (BPE), which was subjected to ^1^H-NMR profiling to carry out fingerprinting of the most abundant metabolites present in the extract, which were related prominently to primary metabolism (Figure 1). The compounds were identified both by their diagnostic signals in the ^1^H NMR spectrum and then further by HSQC and HMBC correlations. 

Indeed, among the carbohydrates, we can count α- and β-glucose, sucrose, and fructose, whose main tautomeric form was β-fructopyranose (β-pyr). It is, in fact, known that at equilibrium in water (at room temperature), β-pyr is the preponderant tautomer, followed by β-fructofuranose (β-fur), and then α-fructofuranose (α-fur), accounting for 69.6%, 21.1%, and 5.7% of the solubilized fructose, respectively [19]. Moreover, in BPE, β-pyr was also found to be the most abundant compound and was semi-quantified in 247.3 µg/mg of extract dried weight (DW).

^1^H-NMR analysis also revealed the presence of aliphatic amino acids such as alanine, valine, leucine, and isoleucine; aromatic amino acids including tyrosine and phenylalanine; as well as polar amino acids such as threonine, γ-amino acids such as GABA, and organic acids such as succinic, citric, fumaric acids, and formic acid. Aromatic signals typical of flavonoids were also detectable (i.e., doublets at δ 6.76; 6.85, HSQC correlating with δ 117.7, 118.9, respectively), but due to the low concentration, their structure could not be fully elucidated. This analysis also revealed the presence of the pyridine alkaloid trigonelline, present in many plant tissues and particularly abundant in legumes. Acting as a modulator of prostaglandin receptors, it is attributed to anti-inflammatory as well as antioxidant properties. It is also known for its appetite-stimulating, hypoglycemic, galactogenic, antispasmodic, immune-stimulating, and diuretic properties.

The concentration of the most abundant compounds found by ^1^H NMR profiling and subjected to semi-quantitative analysis is reported in Table 3.

### 3.3. Characterization of Secondary Metabolites

#### 3.3.1. Preliminary Phytochemical Analyses

Beyond the primary metabolites identified—many of which exhibit noteworthy health-promoting properties—the borlotto bean pod, despite typically being regarded as agricultural waste, represents a promising source of polyphenols.

Table 4 presents the results of the characterization of the major classes of secondary metabolites using in vitro colorimetric assays. This preliminary screening reveals that BPE contains a substantial amount of total phenolic compounds, 556.89 ± 47.91 mg GAE/100 g DE, approximately one-fifth of which are flavonoids: 113.96 ± 8.55 mg QE/100 g DE. The vanillin index assay suggests that a large proportion of these flavonoids belong to the flavan-3-ol subclass, such as catechin, epicatechin, and their derivatives.

A modest concentration of proanthocyanidins—condensed tannins capable of releasing anthocyanidins upon acid hydrolysis—was also detected. However, the calculated polymerization index, expressed as the ratio between flavan-3-ols and proanthocyanidins, indicates that the borlotto bean pod is primarily a rich source of monomeric phenolic compounds.

#### 3.3.2. LC-DAD-ESI-MS/MS Analysis

**Figure 2:**
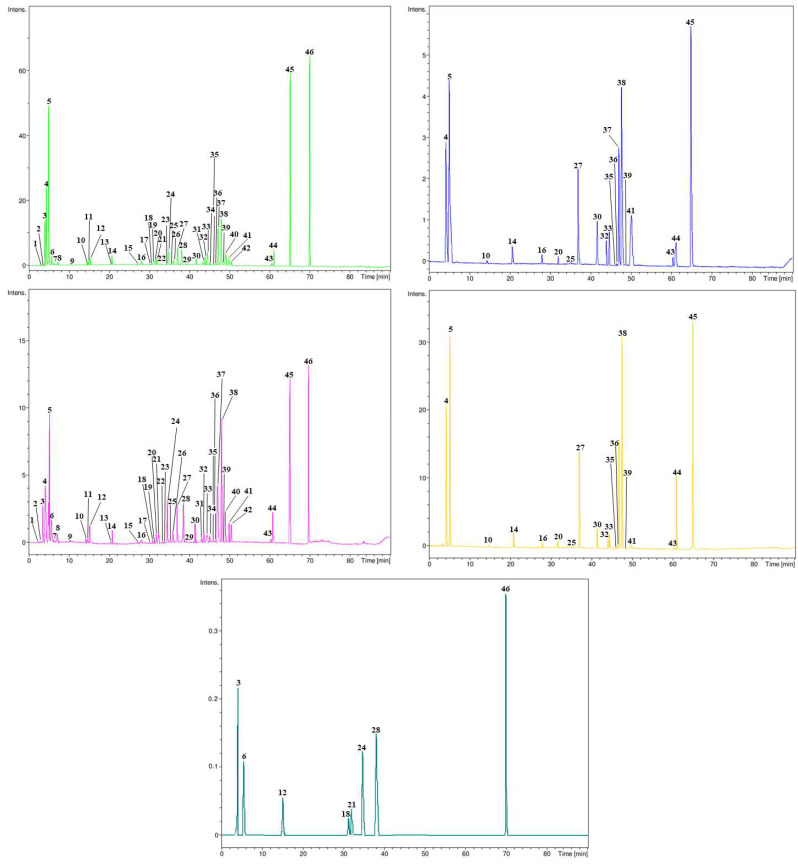
Representative LC-DAD chromatogram of the borlotto bean pod extract (BPE). The chromatogram displays diode array detector (DAD) acquisitions at multiple wavelengths, selected to capture the characteristic absorbance profiles of different polyphenol subclasses: 260 nm (green), 292 nm (pink), 330 nm (blue), 370 nm (yellow), and 520 nm (petrol). Peak numbers correspond to the elution order listed in Table 5.

The LC-DAD-ESI-MS/MS analysis revealed a highly diverse profile of secondary metabolites in the BPE, highlighting its rich phytochemical composition (Table 5 and Figure 2).

A broad range of flavonoids, anthocyanins, phenolic acids, tannins, and other bioactive compounds were identified, underscoring the potential health benefits and biofunctional properties of this agricultural by-product.

Flavonoids were particularly abundant, with quercetin derivatives dominating the profile. Notably, quercetin 7-*O*-glucuronide (peak intensity 19.20 × 10^6^) and quercetin 3-*O*-glucoside (12.13 × 10^6^) were among the most intense signals, confirming a strong presence of glucuronidated and glycosylated quercetin forms, which are known for their antioxidant and anti-inflammatory activities.

Kaempferol and its glycosides were also well-represented, with kaempferol itself showing high abundance (18.40 × 10^6^), followed by its various glicosylated and malonylated forms. Among the anthocyanins, delphinidin 3-*O*-glucoside (9.11 × 10^6^), pelargonidin 3-*O*-glucoside (6.44 × 10^6^), and malvidin 3-*O*-glucoside (1.01 × 10^6^) were identified, reflecting the presence of pigmented compounds that could contribute to both color and antioxidant activity. The strong signal of malvidin (23.21 × 10^6^) further confirms the bean pod’s richness in this class of bioactives. The presence of ellagic acid (22.70 × 10^6^), a potent antioxidant, and hydrolyzable tannins such as tellimagrandin I (5.91 × 10^6^) and punicalin suggests a notable contribution from ellagitannins, which may enhance the BPE’s radical scavenging capacity and health-promoting potential.

Flavan-3-ols, including catechin, epicatechin, and their oligomeric forms such as prodelphinidin B, were present in modest amounts, confirming the results of preliminary colorimetric assays. Several glycosylated and dimeric flavan-3-ol derivatives (e.g., (epi) gallocatechin-dihexoside) were detected, supporting the conclusion that BPE is predominantly composed of monomeric forms, with limited polymerization. Additionally, rare or structurally diverse compounds such as trans-resveratrol disulfates, eucomic acid, and pseudouridine indicate the complexity of the metabolomic profile and suggest potential avenues for further pharmacological investigation. In summary, the LC-DAD-ESI-MS/MS data corroborate the preliminary findings of a high phenolic content in the borlotto bean pod and provide detailed insight into the nature and diversity of its bioactive constituents. These results support the valorization of bean pod waste as a source of functional compounds with potential applications in nutraceuticals, functional foods, or cosmetics.

### 3.4. Antioxidant and Anti-Inflammatory Activities

**Figure 3:**
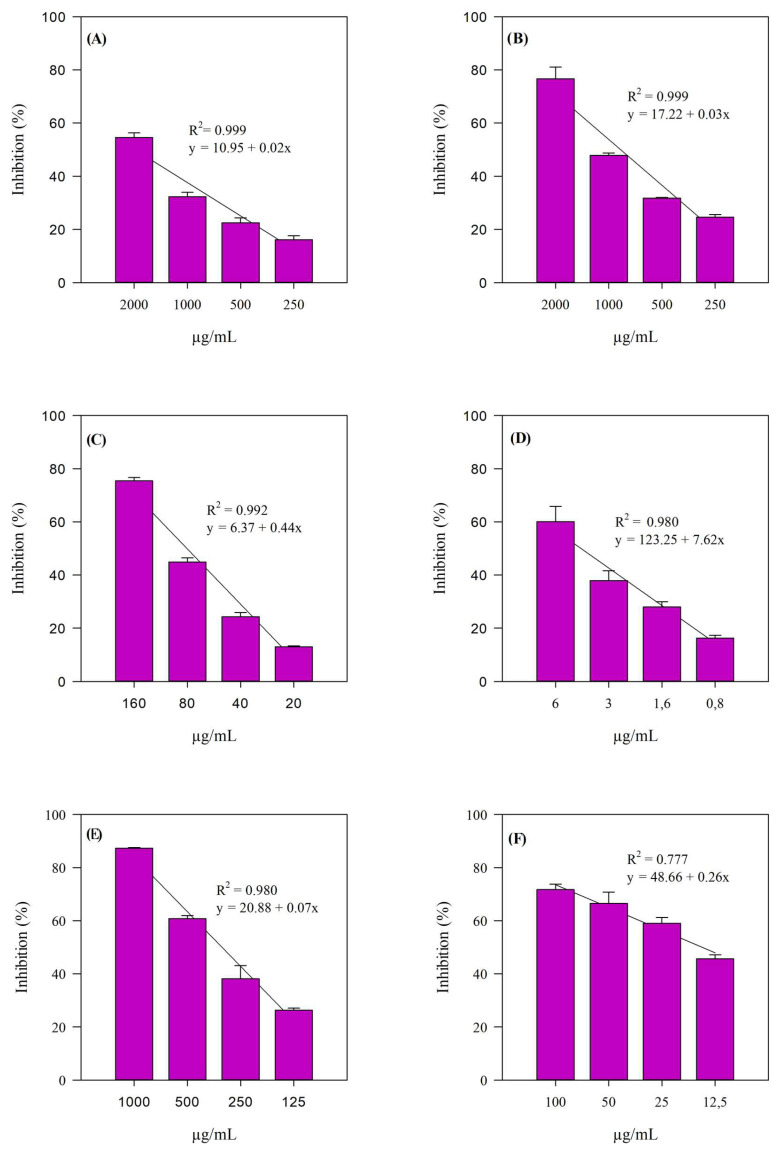
Antioxidant activity of BPE. Data are reported as percentage inhibition (%) and presented as the mean ± standard deviation from three independent experiments conducted in triplicate (*n* = 3). (**A**) DPPH; (**B**) FRAP; (**C**) ICA; (**D**) ORAC; (**E**) TEAC; (**F**) BCB.

To characterize the antioxidant and anti-inflammatory effects of BPE, various in vitro assays, relying on spectrophotometric and fluorometric measurements, were performed. In addition, the concentration-dependent behavior of BPE was evaluated by testing different concentrations and comparing the activity found with that of a suitable reference standard, i.e., a synthetic molecule with proven antioxidant and anti-inflammatory properties. In Figure 3, the results expressed as the inhibition percentage (%) and obtained by studying the free-radical scavenging activity of the extract under examination in different environments and with different reaction mechanisms can be graphically observed: DPPH (A), FRAP (B), ORAC (D), and TEAC (E).

The iron-chelating activity (ICA, panel C), as well as the anti-peroxidative action by means of the β-carotene bleaching test (BCB, panel F), was also evaluated. As can be seen from Figure 2, the extract exhibited consistent linear trends (R^2^ ≥ 0.777) across all assays.

**Figure 4:**
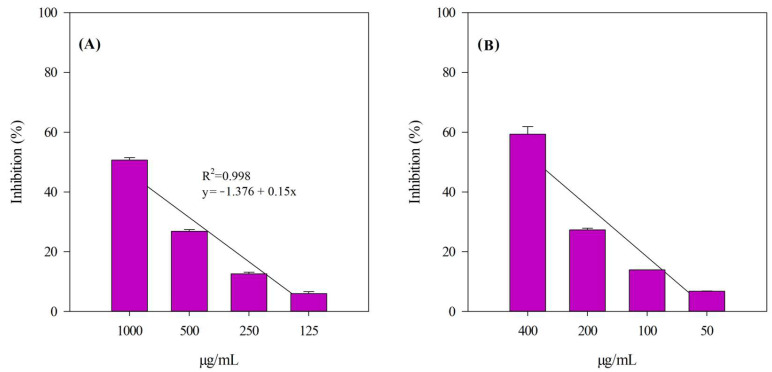
Anti-inflammatory activity of BPE. Percentage inhibition values are presented as the mean ± standard deviation from three independent experiments, each conducted in triplicate (*n* = 3). (**A**) PIA; (**B**) ADA.

The same type of concentration-dependent linear behavior (R^2^ ≥ 0.998) was also found when assessing anti-inflammatory activity, as shown in Figure 4. Specifically, anti-inflammatory activity was evaluated using the protease inhibition assay (PIA, Figure 4, panel (A)) and the heat-induced bovine serum albumin denaturation assay (ADA, Figure 4, panel (B)).

Table 6 shows the results of the antioxidant and anti-inflammatory screening expressed as IC_50_ with the respective 95% C.L.

This also allows an immediate comparison between the activity of BPE and that of the specific reference standard used for each assay (see Table 6 footnote). Although BPE required a higher concentration than the reference standards to achieve comparable effects (*p* < 0.001), it still demonstrated measurable antioxidant and anti-inflammatory activity (Table 6). These findings suggest that BPE exerts its antioxidant activity primarily via hydrogen atom transfer mechanisms (ORAC and BCB), suggesting that its active ingredients act mainly through this mechanism of action in exerting direct antioxidant activity on free radicals. It also showed an excellent iron-chelating capacity, which can certainly be ascribed to its good flavonoid content.

As far as anti-inflammatory activity is concerned, the weaker activity found in the ADA assay suggests that the active ingredients present in the plant complex act more through an enzymatic mechanism, as evidenced in the PIA assay, rather than through an anti-peroxidase mechanism at the expense of membrane proteins.

## 4. Discussion

The food industry produces substantial amounts of waste, which, if not properly managed, can pose significant environmental and public health challenges. Therefore, identifying new applications for these waste products is increasingly prioritized to achieve sustainable development. Waste materials that meet certain technical criteria can be repurposed in various production processes. Such materials include legume pods, fruit peels, and seeds, which are regarded as valuable by-products of the food industry due to their potential as sources of functional compounds [20].

The transformation of biomass into value-added products represents a rapidly growing and evolving field of research. This approach extends beyond food reuse to include applications in nutraceuticals and cosmetics [21]. The common bean (*Phaseolus vulgaris* L.) is one of the most widely cultivated and nutritionally important food crops, valued for its high content of proteins, complex carbohydrates, vitamins, and phenolic compounds [22]. In 2018, bean production in Colombia reached 131,716 tons, while global production amounted to 30.4 million tons [23]. During the post-harvest management of the edible portion, primarily the seeds (beans), significant amounts of waste are generated. The most prominent of these by-products is the pod, which constitutes approximately 39% of the fruit’s weight [24]. Consequently, annual pod production is estimated to be around 84,211 tons in Colombia and 19.4 million tons worldwide.

To the best of our knowledge, this is the first study to examine the nutritional, phytochemical, and biological properties of borlotto bean pods; consequently, no direct comparison with previously published data is currently possible. One notable study investigated bean pod meal using two different drying methods, assessing parameters such as color, water and oil retention capacity, antioxidant capacity (using the TEAC test), polyphenol content, nutritional composition, and morphological characteristics [25].

The study specifically evaluated the impact of drying methods on certain functional properties, such as color, which is a critical attribute for many products incorporating flour, including bread and pasta. The authors found that flours with a yellowish hue and slight red tint exhibited superior quality. Statistically significant differences were noted between drying methods; vacuum-dried samples demonstrated the best quality attributes. Lower drying temperatures minimized matrix browning [26]. However, these differences in color were not statistically significant. These findings are consistent with previous studies on whole grain flours [27] and unconventional flours such as soybean pods [28].

The evaluation of functional properties, including water and oil retention capacities, is essential for determining the potential applications of the flour. These parameters did not vary significantly with drying methods but exhibited higher values (≥7.56 g/g) compared to pea meal (3.69 g/g) and broad beans (4.46 g/g) [29]. These findings are consistent with those of Mateos-Aparicio et al. [30], who reported water-holding capacities of 8.96 g/g and 9.96 g/g for polysaccharides extracted from pea pods and flat green beans, respectively. Furthermore, the obtained values were significantly higher than those recorded for by-products of grapefruit, lemon, orange, and apple (1.6–2.3 g/g) [31], as well as those reported by Masli et al. [32] for by-products of various botanical origins, ranging between 1.3 and 3.9 g/g. The high water-holding capacity of bean pod flour could prove beneficial in products requiring hydration to enhance viscosity, such as baked goods and pasta, or in processes involving mechanical stress, such as extrusion and homogenization, also preventing syneresis during storage [32].

Regarding oil retention capacity, the observed values (≥2.53 g/g) were higher than those found in pea and bean flours (1.14 g/g and 1.42 g/g, respectively) by Belghith-Fendri et al. [29] and those reported for isolated pea (0.28 g/g) and broad bean (0.13 g/g) polysaccharides [30]. They also exceeded values obtained for by-products of orange (2.15 g/g) [33], cauliflower fiber (0.5 g/g) [34], and wheat bran (1.5 g/g) [35], though lower than those of rice husk [36], sugarcane husk (3.26 g/g), and asparagus (5.28–8.53 g/g) [37].

The oil retention capacity is associated with surface properties such as particle size, density, porosity, and hydrophobicity [38]. The high retention values suggest potential applications in the formulation of emulsions and high-fat foods to enhance physicochemical characteristics and flavor retention.

Additionally, the study investigated total phenolic content and antioxidant activity of bean pod flour. Significant differences (*p* < 0.05) were noted between drying methods. Interestingly, flour produced by convective drying exhibited higher antioxidant capacity and total phenol content, contrary to expectations, as polyphenols are typically prone to degradation during heat treatment [39]. This result may be attributed to the formation of additional compounds via the Maillard reaction [40,41], as evidenced by reduced brightness in convectively dried flour [25]. The high phenolic content observed aligns with the known tendency of phenolic compounds to accumulate in plant dermal tissues for photoprotection and defense against pathogens and herbivores [42]. Foods with potent antioxidant properties are highly valued for their potential health benefits [40]. Therefore, bean pod flour shows promise for developing functional foods or nutraceuticals. Overall, the findings by Martínez-Castaño et al. [25] indicate that convective drying is a cost-effective and efficient method for producing bean pod flour while preserving functional properties such as color, water-holding capacity, and oil retention. The obtained flour exhibited high dietary fiber content (66.93%), indicating its potential as a low-fat, mineral-rich ingredient.

## 5. Conclusions

This study provides the first comprehensive nutritional and phytochemical characterization of borlotto bean pods, highlighting their content of dietary fiber and proteins, and revealing a complex phytochemical profile dominated by flavonoids and hydroxycinnamic acids. BPE exhibited clear antioxidant and radical scavenging activity in a concentration-dependent manner, as demonstrated by multiple in vitro assays. The findings suggest that borlotto bean pods, currently considered an agro-industrial by-product, represent a promising source of bioactive molecules with potential nutraceutical value and relevance for circular economy approaches.

Future research should focus on the bioaccessibility and bioavailability of the major phenolic constituents of BPE through in vitro gastrointestinal digestion models and/or in vivo studies, developing food-grade delivery systems for their incorporation into functional products, and evaluating potential health effects in vivo using appropriate animal models or clinical settings. Moreover, future work should also include the absolute quantification of key phenolic compounds using validated external standards to substantiate potential nutraceutical applications and meet regulatory requirements.

## Figures and Tables

**Table 1:** Nutritional characteristics of the borlotto bean pod (*Phaseulus vulgaris* L.). Results, expressed as grams per 100 g of fresh weight (FW), represent the mean ± standard deviation of three independent experiments conducted in triplicate (*n* = 3). Calorific value was expressed both in Kcal and KJ.

Nutritional Value	g/100 g
Calories	69 Kcal–287 KJ
Moisture	77.43
Fats	0.46
Proteins	0.59
Sugars	10.90
Total fiber	9.30
Ash	1.20
Sodium (mg)	9.10

**Table 2:** Characterization of the fatty acids profile of borlotto bean pod (*Phaseulus vulgaris* L.) by GC-FID. Results were expressed as percentages (%) of the total identified and quantified FAME and are presented as the mean ± standard deviation of three independent experiments performed in triplicate (*n* = 3).

Fatty Acids	g/100 g (%)
C12:0-Lauric	0.64 ± 0.08
C14:0-Myristic	1.07 ± 0.17
C15:0-Pentadecanoic	0.34 ± 0.07
C16:0-Palmitic	23.47 ± 1.30
C17:0-Margaric	0.75 ± 0.08
C18:0-Stearic	7.59 ± 0.44
C20:0-Arachidonic	0.85 ± 0.09
C22:0-Behenic	0.25 ± 0.06
C24:0-Lignoceric	0.16 ± 0.06
Saturated fatty acids (SFA)	35.13 ± 1.13
C14:1-Myristoleic	0.36 ± 0.08
C16:1n-9-Trans-palmitoleic	0.66 ± 0.09
C16:1n-7-Cis-palmitoleic	1.16 ± 0.08
C17:1-Heptadecanoic	0.53 ± 0.07
C18:1n-9-Oleic	47.56 ± 2.07
C18:1n-7-Cis-Vaccenic	0.85 ± 0.09
C20:1 n-9-Eicosenoic	0.46 ± 0.09
C24:1 n-9-Nervonic	0.11 ± 0.04
Monounsaturated fatty acids (MUFA)	51.69 ± 2.02
C18:2n-6-Linoleic	10.45 ± 1.02
C18:3n-6-γ-Linolenic	0.57 ± 0.09
C18:3n-3-α-Linolenic	1.29 ± 0.14
C18:4n-3-Stearidonic	0.53 ± 0.10
C18:4n-1-9,12,15,17-octadecatetraenoic	0.34 ± 0.08
Polyunsaturated fatty acids (PUFA)	13.18 ± 1.10

**Table 3:** Semiquantitative analysis of compounds detected by ^1^H NMR analysis. Values are expressed as µg of metabolite/mg of extract dried weight (DW) and in percentage.

	^1^H-NMR Diagnostic Signal (δ, Multiplicity)	µg/mg DW	%
Leucine	0.98, d	10.5	1.1
Isoleucine	1.02, d	7.5	0.7
Valine	1.05, d	7.4	0.7
Threonine	1.33, d	8.6	0.9
Alanine	1.49, d	8.1	0.8
Citric Acid	2.68, d	101.4	10.1
Asparagine	2.94, dd	16.4	1.6
GABA	3.00, t	13.7	1.4
β-Fructopyranose	3.94, m	247.3	24.7
β-Glucose	4.6, d	77.3	7.7
α-Glucose	5.2, d	72.8	7.3
Sucrose	5.4, d	113.5	11.3
Fumaric Acid	6.54, s	3.6	0.4
Tyrosine	7.18, d	8.1	0.8
Phenylalanine	7.37, m	11.3	1.1
Formic Acid	8.46, s	2	0.2
Trigonelline	8.9, m	7.2	0.7

δ = chemical shift, d = doublet dd = double doublet, m = multiplet, s = singlet, t = triplet.

**Table 4:** Determination of the main classes of secondary metabolites of borlotto bean pod extract (BPE). Results are presented as the mean ± standard deviation (S.D.) of three independent experiments, each performed in triplicate (*n* = 3).

Assay	BPE
Total phenols (mg GAE ^a^/100 g DE ^b^)	556.89 ± 47.91
Flavonoids (mg RE ^c^/100 g ES)	113.96 ± 8.55
Flavan-3-ols (mg CE ^d^/100 g DE)	102.82 ± 2.35
Proanthocyanidins (mg CyE ^e^/100 g DE)	5.38 ± 0.27
Polymerization index ^f^	19.11

^a^ GAE, gallic acid equivalents; ^b^ DE, dry extract; ^c^ RE, rutin equivalents; ^d^ CE, catechin equivalents; ^e^ CyE, cyanidin chloride equivalents; ^f^ polymerization index = flavan-3-ols/proanthocyanidins.

**Table 5:** LC-DAD-ESI-MS/MS analysis of borlotto bean pod extract (BPE). The abundance of polyphenols in TIC chromatogram was expressed as ion peak intensity.

Compound	n.	RT ^a^ (min)	λ_max_ (nm)	MW ^b^	Peak Intensity (×10^6^)	[M+H]^+^ (*m/z*)	MS/MS	[M+H] (*m/z*)	MS/MS
Phenylalanyl-leucine	1	2.9	260	278	0.17	-	-	277	233; 130; 146; 120
Raffinose or isomer	2	3.3	205	504	0.13	-	-	503	341; 179; 161; 119
Delphinidin 3-*O*-glucoside (Mirtillin) ^c^	3	3.8	275, 535	465	9.11	465	303; 285; 153	-	-
Quercetin 3-*O*-glucoside (Isoquercitrin) ^c^	4	4.1	260, 365	463	12.13	465	303; 285; 273	-	-
Quercetin 7-*O*-glucuronide	5	5.2	260, 360	478	19.20	479	303; 285; 273	-	-
Pelargonidin 3,5-*O*-diglucoside	6	5.7	270, 515	631	4.21	632	469; 307; 289	-	-
Pseudouridine	7	5.8	260	244	0.21	-	-	243	113; 95; 132; 211
Punicalin A or B	8	7.1	270	782	1.46	783	613; 481; 301; 171	-	-
(epi)gallocatechin-(epi)catechin II	9	10.3	278	594	0.09	-	-	593	425; 289; 305; 407
Kaempferol-3,7-diglucoside	10	14.3	270, 360	610	0.17	-	-	609	447; 285; 151; 133
(epi)gallocatechin-(epi)catechin I	11	14.5	278	594	0.17	-	-	593	425; 305; 289
Petunidin ^c^	12	14.9	275, 530	317	2.58	318	302; 287; 273; 153	-	-
(epi)gallocatechin-dihexoside	13	20.5	278	630	0.16	-	-	629	467; 305; 287; 179; 151
Quercetin 3-*O*-rutinoside (Rutin) ^c^	14	20.6	260, 358	610	2.70	611	465; 303; 285; 229	-	-
(epi)catechin hexoside	15	26.9	278	452	0.11	-	-	451	289; 271; 245; 151; 125
Quercetin 3-*O*-(6″-*O*-malonyl)glucoside	16	27.9	260, 368	550	1.35	551	465; 303; 285; 229	549	463; 301; 179; 151
(epi)gallocatechin-*O*-hexoside	17	30.2	273	468	0.09	-	-	467	287; 179; 151
Malvidin 3-*O*-glucoside ^c^	18	30.9	270, 530	493	1.01	494	331; 316; 301; 153	-	-
Ciceritol	19	31.1	205	518	0.10	-	-	517	355; 193; 179; 161; 149
Kaempferol 3-*O*-glucoside (Astragalin)^c^	20	31.8	265, 350	448	1.12	449	287; 269; 153; 121	-	-
Cyanidin 3-*O*-(6″-malonyl)glucoside	21	31.9	270, 530	535	1.16	535	449; 287	-	-
Uralenneoside	22	33.0	260	286	0.11	-	-	285	153; 267; 249; 231
(epi)catechin-*O*-dihexoside	23	34.1	278	614	0.11	-	-	613	451; 289; 245; 203
Pelargonidin 3-*O*-glucoside ^c^	24	34.5	270, 510	433	6.44	433	271; 253; 153; 137	-	-
Quercetin 3,4′-diglucoside	25	35.0	260, 350	626	0.13	-	-	625	463; 301; 271; 255
Eucomic acid	26	35.9	275	240	1.52	-	-	239	179; 149
Myricetin 3-*O*-glucoside ^c^	27	37.3	260, 365	480	8.30	481	319; 301; 179; 151	479	317; 299; 271; 179; 151
Pelargonidin 3-*O*-(6″-malonyl)glucoside	28	38.2	270, 515	519	7.23	519	433; 357; 255	-	-
Catechin ^c^	29	39.3	278	290	0.11	-	-	289	245; 205; 179; 151
Kaempferol 3-*O*-(6″-*O*-malonyl)glucoside	30	41.7	260, 360	534	3.38	535	449; 271; 153; 121	-	-
Prodelphinidin B	31	43.5	280	610	1.05	-	-	609	483; 441; 305
Kaempferol 3-*O*-(malonyl)glucoside	32	43.9	260, 360	534	2.47	535	449; 271; 153; 121	-	-
Quercetin 3-*O*-xylosylglucoside	33	44.4	260, 355	596	3.02	597	465; 303; 285; 229; 153	595	463; 301; 271; 255; 179
Taxifolin hexoside	34	45.1	287	466	1.26	-	-	465	303; 285; 275; 151; 125
Kaempferol-glucoside-rhamnoside	35	46.1	260, 350	594	0.20	-	-	593	447; 431; 285; 257; 151
Kaempferol 3-*O*-rhamnoside	36	46.7	260, 350	432	0.64	-	-	431	285; 151
Myricetin ^c^	37	47.2	265, 365	318	10.58	319	301; 291; 275; 179; 153	-	-
Kaempferol ^c^	38	47.7	260, 360	286	18.40	287	269; 259; 153; 121	-	-
(epi)catechin-phloroglucinol	39	48.2	270, 325	414	0.14	-	-	413	289; 125; 245; 151
Tellimagrandin I	40	49.3	275	786	5.91	787	617; 465; 301; 169	-	-
trans-Resveratrol-disulfate	41	49.8	307, 321	388	4.75	389	309; 229; 211	-	-
Benzoic acid-3-glucuronide-4-sulfate	42	50.2	215, 240	410	4.16	411	231; 193; 113	-	-
Luteolin ^c^	43	60.4	265, 345	286	0.80	-	-	285	257; 241; 217; 175; 151
Kaempferol 3-*O*-xylosylglucoside	44	60.8	260, 350	580	5.96	581	449; 287; 269; 153	-	-
Ellagic acid ^c^	45	65.0	254, 362	302	22.70	303	285; 257; 229; 185	-	-
Malvidin ^c^	46	69.9	270, 530	331	23.21	331	316; 313; 299; 271	-	-

^a^ RT, retention time; ^b^ MW, molecular weight; ^c^ checked with commercially available HPLC-grade reference standards (purity ≥ 98%) purchased from Extrasynthase (Genay, France) or Merck KGaA (Darmstadt, Germany).

**Table 6:** Determination of antioxidant and anti-inflammatory properties of BPE. Results are expressed as the concentration required to inhibit 50% of the oxidative or inflammatory activity (IC_50_), along with the corresponding 95% confidence limits (C.L.), and represent the mean of three independent determinations conducted in triplicate (*n* = 3).

Assay	BPE	Standard ^a^
	IC_50_ µg/mL (C.L. ^b^ 95%)	
DPPH	1878.78 (1384.50–2549.51)	15.99 (12.92–19.81)
FRAP	702.890 (297.48–1660.78)	3.64 (1.76–7.52)
TEAC	248.5 (235.32–343.97)	4.34 (2.27–8.33)
ORAC	4.06 (1.85–8.93)	0.72 (0.02–1.84) ***
ICA	83.90 (69.74–100.95)	5.78 (3.03–11.03) ***
BCB	31.15 (22.17–43.74)	0.37 (0.15–0.76) ***
ADA	1035.88 (854.34–1256.71)	60.81 (38.93–94.99) ***
PIA	370.560 (296.34–463.38)	23.23 (13.93–38.73) ***

^a^ Trolox for DPPH, FRAP, TEAC, and ORAC, EDTA for iron chelating activity (ICA), and BHT for β-carotene bleaching (BCB); Diclofenac sodium for ADA and PIA ^b^ 95% confidence limits calculated with Litchfield and Wilcoxon test using PHARM/PCS software (version 4; Consulting, Wynnewood, PA, USA). *** *p* < 0.001 vs. BPE.

## Data Availability

The original contributions presented in the study are included in the article; further inquiries can be directed to the corresponding author.

## Abbreviations

The following abbreviations are used in this manuscript:

ABTS	2,2′-Azino-bis-(3-ethylbenzothiazoline-6-sulfonic acid)
ADA	Albumin Denaturation Assay
AAPH	2,2′-Azobis(2-amidinopropane) dihydrochloride
BHT	Butylated Hydroxytoluene
BPE	Bean Pod Extract
BSA	Bovine Serum Albumin
BCB	β-Carotene Bleaching
CD_3_OD	Deuterated Methanol
C.L.	Confidence Limits
D_2_O	Deuterium Oxide
DPPH	2,2-Diphenyl-1-picrylhydrazyl
DW	Dry Weight
EDTA	Ethylenediaminetetraacetic Acid
ESI	Electrospray Ionization
FAME	Fatty Acid Methyl Esters
FID	Flame Ionization Detector
FRAP	Ferric-Reducing Antioxidant Power
GAE	Gallic Acid Equivalents
GC	Gas Chromatography
HMBC	Heteronuclear Multiple Bond Correlation
HSQC	Heteronuclear Single Quantum Coherence
ICA	Iron-Chelating Activity
IC_50_	Half Maximal Inhibitory Concentration
ICP-MS	Inductively Coupled Plasma–Mass Spectrometry
LC-DAD-ESI-MS	Liquid Chromatography with Diode Array Detection and Electrospray Ionization Mass Spectrometry
MES	2-(N-Morpholino)ethanesulfonic Acid
MUFA	Monounsaturated Fatty Acids
NMR	Nuclear Magnetic Resonance
ORAC	Oxygen Radical Absorbance Capacity
PBS	Phosphate Buffered Saline
PIA	Protease-Inhibitory Activity
PUFA	Polyunsaturated Fatty Acids
QE	Quercetin Equivalents
RE	Rutin Equivalents
RT	Room Temperature
SFA	Saturated Fatty Acids
TEAC	Trolox Equivalent Antioxidant Capacity
TMSP	3-(Trimethylsilyl)-propionic Acid-2,2,3,3-d_4_
TRIS	Tris(hydroxymethyl)aminomethane
TPTZ	2,4,6-Tris(2-pyridyl)-s-triazine
UV-vis	Ultraviolet-visible
